# A pilot animal and clinical study of autologous blood solution compared with normal saline for use as an endoscopic submucosal cushion

**DOI:** 10.3892/etm.2012.626

**Published:** 2012-06-29

**Authors:** WEI WEN, CHUANBING SHI, YAN SHI, GUOZHONG JI, PING WU, ZHINING FAN, FAMING ZHANG

**Affiliations:** 1 Institute of Digestive Endoscopy and Medical Center for Digestive Diseases, The Second Affiliated Hospital of Nanjing Medical University, Nanjing 210011, P.R. China; 2 Department of Pathology, The Second Affiliated Hospital of Nanjing Medical University, Nanjing 210011, P.R. China

**Keywords:** autologous blood, tissue damage, endoscopic submucosal dissection, endoscopic musocal resection, artificial ulcer

## Abstract

Normal saline is the most popular agent used during endoscopic submucosal injection. However, endoscopists have never identified an optimal submucosal injection solution, which is not only safe and cost-effective but has a unique lifting ability with endoscopic submucosal cushion and causes less tissue damage. This study aimed to evaluate the effectiveness and microscopic characteristics of a blood solution, including whole blood and plasma solution, as a submucosal cushioning agent, compared with normal saline. Endoscopic submucosal dissection (ESD) procedures in pig stomachs were performed by injecting plasma solution (n=4) and normal saline (n=4). A total of 38 patients with gastrointestinal neoplasms underwent endoscopic musocal resection (EMR) procedures. Of 38 EMRs, 7 used whole blood injection, and 31 of 38 acting as the control group used normal saline. A tissue damage scoring system was developed based on injection-induced hydrops and tears for the evaluation of tissue damage. In animal experiments, the lifting time of the injection with normal saline in the pig colon was shorter than that of the group with plasma solution (18.25±5.44 min vs. 6.5±2.38 min, P=0.007). In animal experiments with ESD procedures in the stomach, the hydrops in the normal saline injection group were more extensive than those in the group with plasma (P=0.011). The degree of tearing in the group with normal saline was observed to be less than that in the group with plasma (P=0.008). In patients with EMR, using the histological scoring method, it was determined that the degree of hydrops in the group with normal saline injection was more extensive than that in the group with whole blood (P<0.001). The effective submucosal tearing in the group with normal saline was less than that in the group with blood (P<0.001). The blood solution, including whole blood and plasma solution, as a novel submucosal injection agent, may outperform normal saline with a unique lifting ability, less pronounced tissue damage and marked effective submucosal blunt dissection.

## Introduction

Submucosal injection assisted endoscopic mucosal resection (EMR) and endoscopic submucosal dissection (ESD) have been widely used in the removal of benign and early malignant lesions of the gastrointestinal tract (1). EMR, ESD and peroral endoscopic myotomy (POEM) (2) have inspired endoscopists and endoscopic surgeons to identify an optimal submucosal injection solution, which is not only safe and cost-effective but has a unique lifting ability with endoscopic submucosal cushion and aids the early healing of artificial ulcers (3,4).

In clinical practice, a number of cushioning agents are used for endoscopic submucosal injection (5–7). At present, normal saline is the most popular agent used during EMR, ESD and POEM. However, since normal saline absorbs too quickly (8,9), repeated injections during surgical procedures may be necessary to maintain effective lifting. One of the problems is that more injections may result in a greater cost of anesthesia and nursing. The most significant issue is that there is no report regarding an understanding of injection-caused submocosal tissue damage, which may play a key role in the early healing of artificial ulcers.

Hyaluronic acid, glycerol, hydroxypropyl methylcellulose and sodium alginate appear to have the most durable cushioning effect (5,7,10–12). These agents with durable cushioning effects are not only quite expensive but are also difficult to inject (13). Additionally, a concern with hyaluronic acid is the stimulation of tumor cells (14,15), and hydroxypropyl methylcellulose has the ability to cause a local inflammatory reaction and could potentially give rise to antigenic reactions at high doses (11,16). Previous human and animal studies (13,17,18) demonstrated that autologous whole blood produced the most durable cushion compared with standard agents. The autologous blood was noted to have the advantages of being readily available and cost-free. However, there were no systemic studies on tissue damage following ESD or EMR using whole blood and plasma, since the plasma may have different advantages for submucosal injection.

Notably, although the risk factors for bleeding and artificial ulcer healing following ESD using normal saline as a cushioning agent in patients with gastrointesintal neoplasm have been studied in previous studies (19), tissue damage during therapy has not been analyzed as an independent factor. In the present study we assumed that submucosal injection with normal saline induced tissue damage, and may affect the artificial ulcer healing following ESD and EMR. Therefore, the authors designed the animal and human studies to investigate the endoscopic and microscopic characteristics of a bood solution as a cushioning agent and provide a more extensive understanding of submucosal injection-caused tissue damage. In order to demonstrate the advantages of autologous whole blood or plasma solution as a submucosal injection agent, the most comonly used normal saline was used as the control for comparison.

## Materials and methods

### Animals, patients and materials

Four minipigs (Wuzhishan minipig, mean weight 15 kg) were obtained from the Jiangsu Academy of Agricultural Science (Nanjing, China). The animal preparation prior to endoscopy and the management of the animals after the endoscopic procedure were carried out by trained veterinarians. General anesthesia with endotracheal intubation was administered. The endoscopy was carried out with standard endoscopes (GIF-Q240 and CF-260, Olympus, Tokyo, Japan) in the animal laboratory of the Institute of Digestive Endoscopy at the Second Affiliated Hospital (Nanjing Medical University, Nanjing, China) and the animal study was approved by the institutional ethics board.

A total of 38 patients (age 54.37±13.27 years; 25 male, 13 female; 35 with polyps, 2 with early cancer and 1 with cystic gastritis; diameter of neoplasms 12.24±7.1 mm; 4 EMRs in esophagus, 9 in stomach, 1 in duodenum and 24 in large intestine) consented to the EMR procedure of the colon for polyps (between June 2011 and October 2011; Medical Center for Digestive Diseases, Second Affiliated Hospital, Nanjing Medical University, Nanjing, China). Of 38 patients, 7 accepted autologous whole blood submucosal injection for EMR. Of 38 patients, 31 acted as a sham control group and accepted normal saline injection. The human study was approved by the ethics committee of the Second Affiliated Hospital, Nanjing Medical University, Nanjing, China.

### Injection solutions and endoscopic procedures

The normal saline soution for submucosal injection in the animal and human studies was mixed with 1% epinephrine and 0.5% methylene blue. Whole blood was extracted using a vacuum tube, which had 3.2% sodium citrate for anticoagulation (Improve Medical Instruments Company, Guangzhou, China). Using a 23-gauge sclerotherapy needle (Boston Scientific Microvasive, Natick, MA, USA), at least 3 ml of whole blood plus 0.5% methylene blue was injected into the mucosa immediately after it was taken out from the vein. With regard to the experiments in animals, after centrifugation of whole blood at 3,000 rpm for 2 min, the plasma was suctioned from the vacuum tube and mixed with equal volumes of normal saline. A total of 50% pig plasma solution (volume ratio of plasma:normal saline is 1:1) plus 1% methylene blue was prepared for submucosal injection. The volume of plasma solution or normal saline solution for each group was 3 ml in pigs.

The pig colons were used as the organ for evaluation of the lifting effects of the plasma solution and normal saline. The injections in pig colons were administered 4 cm apart, starting above the transverse colon and moving proximally towards the sigmoid colon. Each site of blood injection was closely followed with a normal saline injection for comparison on closer observation. If the mucosa did not elevate after 0.5 ml of the injection, the needle was repeatedly reinserted at different angles until a visible elevation of mucosa was created (13). The time for the mucosal cushion to disappear was recorded. Subsequent injections at the next separate site were performed after the disappearance of the cushion.

The pig stomachs were chosen as the targeting organ for ESD. The mucosal dissections in pig stomachs were performed every 3 cm, starting from the gastric antrum and moving proximally towards the gastric fundus. The ESD procedures were performed for each mucosal dissection (20 mm diameter) and the procedure time from the inception of the submucosal injection to the end of managing the wound surface was recorded. In total, 2 ESDs by the injection of plasma solution and 2 ESDs by the injection of normal saline were performed in each pig stomach. The ESD techniques were performed using a conventional method. In brief, a flex-knife (KD-630L; Olympus, Japan) was used as the main knife for mucosal cutting (dry cut mode, 40 W). All ESD procedures were performed by a single endoscopist. The specimens for haematoxylin and eosin (H&E) staining were placed in a formalin solution immediately after ESD. Endoscopy for follow-up was performed one month later.

### Histological scoring evaluation for tissue damage

A sufficient histological scoring system for evaluating tissue damage following submucosal injection does not exist. In order to evaluate the characteristics of the tissue damage of samples by EMR and ESD, the authors established the histological scoring method for the evaluation of tissue damage following submucosal injection (Table I).

### Statistical analysis

The data were analyzed with statistical software (SPSS v.11.5; SPSS Corp., Chicago, USA). The Mann-Whitney U test was used for nonparametric data of histological scores. The Student’s t-test was used for analysis of the lifting time and procedure time. P<0.05 was considered to indicate a statistically significant result.

## Results

### Surgical procedure

**Figure 1:**
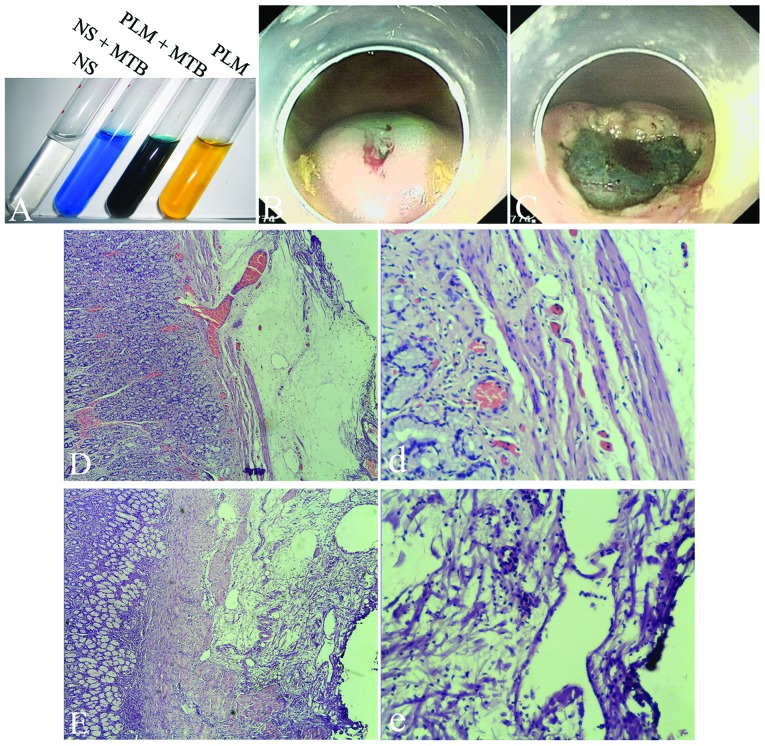
ESD procedures in pig stomachs and tissue damage evaluation following ESD. (A) Different color of injection agents; NS, normal saline; MTB, methylene blue; PLM, plasma. (B) Submucosal injection with plasma. (C) After ESD by plasma injection. (D and d) Marked hydrops and hyperemia were observed from submucosal to mucosa surface after submucosal injection with normal saline. (E and e) Marked demarcated separation of the mucosa and the muscularis propria and uniform distribution (or tears) in the loose submucosa were observed following injection with 50% plasma solution. Magnification: D and E, ×100; d and e, ×400. ESD, endoscopic submucosal dissection.

Submucosal injection in animal colons, ESDs in animal and EMRs in patients were performed easily and no complications were observed. No blood clotting was observed prior to or during injection, but the sclerotherapy needle was partially obstructed 5 min later during whole blood injection. Authors presumed that it was the plasma, not the blood cells, that played the key role in producing effective submucosal seperation and reducing tissue hydrops. The animal experiments were designed to demonstrate the endoscopic and microscopic characteristics of plasma as a lifting agent (Fig. 1).

### Lifting time

The injections with the plasma solution in the submucosa of each pig colon (n=4) were paired with normal saline injections (n=4). The lifting time of the plasma solution (18.25±5.44 min) in pig colons was significantly longer than that in the normal saline group (6.5±2.38 min; t=3.96, P<0.007). However, there was no difference in ESD surgery time between the groups injected with plasma and normal saline in the pig stomachs (7.0±2.12 vs. 5.25±0.96 min, P>0.05; Table II, figures are not shown).

### Animal hydrops and tearing

The hydrops in the normal saline group were more extensive than those in the plasma solution-injected group (P=0.011). Additonally, the tearing in the normal saline group was less than that in the plasma-injected group (P=0.008; Table II and Fig. 1). The animals that underwent ESDs survived and the endoscopy one month later showed the stomach with four well-healed scars (figures not shown).

### Patient hydrops and tearing

**Figure 2:**
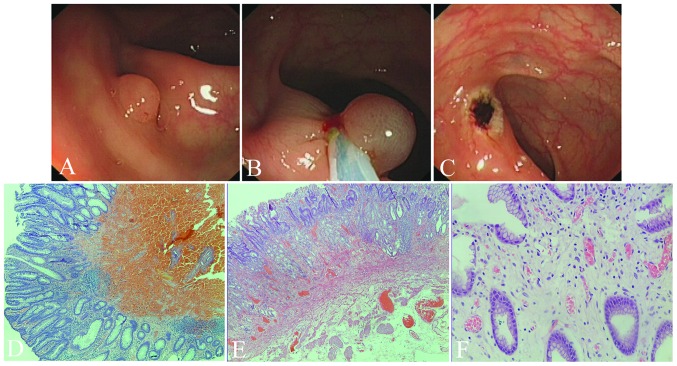
EMR using autologous whole blood in humans. (A) A polyp in the transverse colon. (B) After submucosal injection with 3 ml of autologous blood, the lifted lesion with blood cushion being removed using a snaring technique. (C) Approximately 2 min after the resection, the lifting of mucosa surrounding the site of EMR was not observed. No bleeding occurred during the following 3 min observation. (D) Hematoxylin and eosin (H&E) staining showed that the blood patch within loose submucosa following EMR (C) caused separation of mucosa and muscularis propria, but without tissue damage in the mucosa above the hematoma. (E and F) Serious tissue damage (evaluated as degree 3 for tearing and hydrops, and hyperemia) following EMR using normal saline cushion. Magnification: D and E, ×40; F, ×200. EMR, endoscopic musocal resection.

In order to further demonstrate the endoscopic characteristics of autologous blood injection and the tissue damage following submucosal injection in humans, 38 patients who underwent EMR were studied with the comparison of autologous blood and normal saline. As shown in Fig. 2, Table I and Table III, the hydrops in the group with the normal saline injection were more extensive than those in the group with whole blood (P<0.001). Additonally, the tearing in the group injected with normal saline was less than that in the group injected with blood (P<0.001).

## Discussion

Since the first description in the 1950s of a submucosal injection to assist polypectomy (20), several attempts have been made to find the optimal agent. Such an optimal agent for submucosal injection should be cost-effective, readily available, easy to inject and provide a durable cushion with minimal damage of the surrounding tissues at the site of injection. Previous studies have shown that the use of hyaluronic acid, hydroxypropyl methylcellulose or glycerol provides dextrose water, albumin and sodium alginate, and have a lasting elevating effect (6,7,10,11,21,22). A recent study reported that carbon dioxide (CO_2_) could be a satisfactory submucosal injection agent during ESD (21). The drawbacks of these agents are that they are expensive and difficult to prepare, store or inject (1,3,6,7,10,11,21,23). An additional concern discouraging the use of hyaluronic acid is its potential to stimulate tumor growth, both *in vivo* and *in vitro* (14,15), and the concern with regard to hydroxypropyl methylcellulose is its potential to cause a local inflammatory reaction and give rise to antigenic reactions at high doses (11,16).

The osmolality of the cushioning agents is also another significant determinant in the properties of the injected solutions. Fujishiro *et al* (23) demonstrated that the use of hypertonic solutions (3.75% NaCl and dextrose water at concentrations of 20%) caused significant tissue damage that was not limited to the superficial layers but also extended into the muscularis propria. Bures *et al* (3) reported that viscous or hypertonic solutions for submucosal injection, outperform normal saline and perform as well as sodium hyaluronate in porcine stomachs *in vitro*.

Previous human and animal studies (13,17,18) demonstrated that autologous blood produces the most durable cushion compared with standard agents. Autologous blood also has the advantages of being readily available and without cost. The endoscopic findings further confirmed the durability of whole blood and further demonstrated the durability of plasma solution. The H&E staining in the present study showed whole blood or plasma solution injection within loose submucosa caused separation of the mucosa and muscularis propria layer, but without tissue damage in the mucosa above the hematoma. The current histological supporting evidence from the *in vivo* model demonstrated that blood solution as a cushioning agent causes less tissue damage than normal saline.

The established *in vivo* model is one of the strengths of the present study. The majority of previous studies are based on *ex vivo* studies (3,5,17). The frequently used model for evaluation of injection-induced tissue damage is to inject a solution into the isolated stomach or to resect the targeted stomach wall following injection, instead of gathering tissue samples following the *in vivo* ESD procedure. The disadvantage of an *ex vivo* study is that the time for the submucosal fluid to resolve is prolonged due to the absence of perfusion and active absorption (8). In order to establish a more useful *in vivo* animal model and reduce the number of animals used, due to the high perforation rate of ESD in the colon (19), the colon was used for the evaluation of elevation duration following submucosal injection and stomachs were used for ESD.

The major finding in the present study was that the results demonstrated evidence of tissue damage following submucosal injection with normal saline and blood solution. The fundamental concern of tissue damage is the healing of artificial ulcers and bleeding following mucosa removal (3,4). A tissue damage scoring system was developed based on injection-induced hydrops and tears for the evaluation of tissue damage. The results showed that the blood solution produced a marked blunt dissection and tears of the mucosa from the muscularis propria layer as compared to normal saline. The fundamental principle of submucosal injection for EMR, ESD, POEM and submucosal tunneling endoscopic resection (STER) (2,24,25) is the elevation of targeted tissue or separation of deeper layers in the gastrointestinal tract wall prior to resection, dissection or seperation of targeted tissue. Whole blood and plasma solutions induced tears and may be effective in avoiding possible unpredictable devices assisting separation or sharp dissection causing deeper tissue damage, vessel damage and perforation. This type of effective submucosal tear was first reported by Sumiyama *et al* (26) in 2007 with a cap-fitted endoscope. This technique of blunt dissection/dilation was highlighted in application of natural orifice translumenal endoscopic surgery (NOTES) (27). Due to the fact that normal saline is rapidly absorbed, the injected saline diffuses into the surrounding area of the injection sites and causes serious hydrops and hyperemia, which would delay the early healing of the artificial ulcers and even increase the risk of post-ESD or post-EMR bleeding. Our findings indicate that an ideal submucosal injection solution or plasma-like gel should be transparent, easily injected, nonabsorbing or less absorbable and easily inducing blunt dissection.

During the experiments using the blood solution, we noted that a 23-gauge sclerotherapy needle showed no difficulty in the injection of blood solution, despite the higher injection pressures required, compared with normal saline. Additionally, since gathering blood and centrifugation for plasma are necessary steps for preparing plasma solution, it would take 3 to 5 min more to prepare the injection solution in clinical practice, compared with using normal saline.

Unlike the procedures for varied human lesions, the time of ESD operation in pigs for both groups was short due to the fact that the stomach is normal, instead of in early cancer stages. There was no significant difference between the two groups of ESDs in pig stomachs, but we suggest that the potential difference should be significant in human, since shorter injecting times during ESD would benefit from the much longer lifting time when using plasma solution, instead of normal saline.

With regard to the limitations of this study, a number of hypertonic solutions should be included as control groups, although previous research revealed that the blood provides the longest duration of the submucosal cushion compared with hydroxypropyl methylcellulose, albumin and normal saline (13).

In conclusion, this is the first study using autologous plasma solution as a gastrointestinal submucosal injection agent. The *in vivo* animal and human study demonstrated that whole blood or plasma solution may outperform normal saline due to its unique lifting ability, less tissue damage and marked effective submucosal blunt dissection. The study highlighted that normal saline used as a submucosal injection caused tissue damage, which may affect the early healing of artificial ulcers and be associated with bleeding following mucosal therapies. Our comparative studies are ongoing in order to confirm the clinical benefits of using autologous blood solution for patients with submucosal injection procedures.

## Figures and Tables

**Table I:** Histological score (H-score) for gastrointestinal tissue damage evaluation.

	H-score for tissue damage evaluation	
Parameters	Score	Description
Degree of hydrops	1	Without or only with hydrops in observed SM
	2	Marked hydrops in SM and LMM
	3	Marked hydrops in SM, LMM and ML
Degree of tears	1	Tears exist in <1/3 area of observed SM
	2	Tears exist in 1/3–2/3 area of observed SM
	3	Tears exist in >2/3 area of observed SM

SM, submucosa; LMM, lamina muscularis mucosae; ML, mucous layer. Hyperemia, as one of common tissue damage parameters, was not evaluated in the present study.

**Table II:** Tissue damage evaluation for tissues dissected by ESD in the pig stomachs.

		H-score of hydrops				H-score of tears			
Group	n	1	2	3	P-value^a^	1	2	3	P-value^a^
Normal saline	4	0	0	4	0.011	4	0	0	0.008
Plasma solution	4	3	1	0		0	0	4	

a Mann-Whitney U test. H-score, histological score; ESD, endoscopic submucosal dissection.

**Table III:** Tissue damage evaluation for tissues dissected by EMR in humans.

		H-score of hydrops				H-score of tears			
Group	n	1	2	3	P-value^a^	1	2	3	P-value^a^
Normal saline	31	0	3	28	<0.001	29	2	0	<0.001
Blood solution	7	7	0	0		0	1	6	

a Mann-Whitney U test. EMR, endoscopic musocal resection; H-score, histological score.
